# The Role of Selected Lactic Acid Bacteria on Organic Acid Accumulation during Wet and Spray-Dried Fish-Based Silages. Contributions to the Winning Combination of Microbial Food Safety and Environmental Sustainability

**DOI:** 10.3390/microorganisms8020172

**Published:** 2020-01-25

**Authors:** Esmeray Kuley, Gulsun Özyurt, Ilyas Özogul, Mustafa Boga, Ismail Akyol, João M. Rocha, Fatih Özogul

**Affiliations:** 1 Department of Seafood Processing Technology, Faculty of Fisheries, Cukurova University, Balcali, 01330 Adana, Turkey; eboga@cu.edu.tr (E.K.); beklevik@cu.edu.tr (G.Ö.); fozogul@cu.edu.tr (F.Ö.); 2 Imamoglu Vocational School, Cukurova University, 01330 Adana, Turkey; iozogul@cu.edu.tr; 3 Bor Vocational School, Nigde Omer Halisdemir University, 51700 Nigde, Turkey; mboga@ohu.edu.tr; 4 Department of Animal Science, Faculty of Agriculture, University of Ankara, 06110 Ankara, Turkey; iakyol@ankara.edu.tr; 5 REQUIMTE—Rede de Química e Tecnologia, Laboratório de Química Verde (LAQV), Departamento de Química e Bioquímica, Faculdade de Ciências da Universidade do Porto (FCUP), Rua do Campo Alegre, s/n. P-4169-007 Porto, Portugal; 6 ICETA - Instituto de Ciências, Tecnologias e Agroambiente da Universidade do Porto, Praça Gomes Teixeira, Apartado 55142, 4051-401 Porto, Portugal

**Keywords:** fish silage, fish residues or by-products, fermentation, lactic acid bacteria, organic acids, lactic acid, acetic acid, formic acid, succinic acid, propionic acid, microbiota and microbial safety, microbial starter cultures, food safety, food preservation, recovery of by-products, environmental sustainability

## Abstract

Organic acid contents of acidified and fermented fish silages made from gibel carp (*Caracius gibelio*) and klunzinger’s ponyfish (*Equulites klunzingeri*) fishes, and from fish processing residues or by-products, were determined and studied. The silages were undertaken in wet and spray-dried fish-based raw-materials for 3 weeks at room temperature (*ca*. 25 °C). Selected lactic acid bacteria (LAB) of *Enterococcus gallinarum*, *Lactobacillus brevis*, *Lactobacillus plantarum*, *Pediococcus acidilactici,* and *Streptococcus* spp. were employed to produce fermented fish-based silages, while acidified silage was prepared resorting to the addition of formic acid (3%, v/v). Lactic and propionic acids were the dominant produced organic acids, while succinic acid was formed at the smallest amounts in fermented silages. In the acidified silage, lactic and formic acids were produced in amounts higher than 800 and 1000 mg _organic acid_/100 g _sample_, respectively. Among the fermented fish-based silages, LAB strains unfolded considerable ability to presumptively produce propionic acid in gibel carp silage (>2370 mg _organic acid_/100 g _sample_). Spray-dried fermented silages displayed significantly higher organic acid content than wet silages. Propionic acid accumulation was found at the highest levels in gibel carp silage fermented with *L. plantarum* (6335.40 mg _propionic acid_/100 g _sample_). This research effort pointed out the good capability of various selected lactic acid bacteria strains to produce significant amounts of organic acids—especially lactic, acetic, and propionic acids—during the fermentation of fish-based silages. In terms of food safety and quality, such a production of relatively high amounts of organic acids in wet and spray-dried fish-based silages clearly indicated their suitableness to be used for animal feed.

## 1. Introduction

The fish processing industry generates a large amount of high quality protein food residues or by-products. The term by-product or residual (not residue) is preferred to the term waste in the context of the circular economy for environmental sustainability. When treated as disposable wastes and not as recoverable by-products for other purposes, this material represents a source of serious environmental pollution and/or additional costs related with landfilling or composting. It was reported that approximately 63.6 million tons of fish waste is generated globally, based on the assumption that ca. 45% of the weight of live fish is not used and eventually originates waste [1,2].

Gibel carp (*Caracius gibelio*) and ponyfish (*Equulites klunzingeri*) are examples of fish species without effective economic value. Silage is recognized as being the most useful biotechnology for solving the utilization of discarded (no economic value) fish as well as the disposable fish-based wastes in the fish processing industry. Besides representing a high-value product for its protein content, the production of fish silages is a preferred processing biotechnology because it requires low inputs of energy, labor, and equipment. Therefore, fish without commercial value and fish-based wastes from the processing industry can be converted favorably into powdered/flour fish silage, since it is a relatively easy method to be implemented and provides relatively low operating and initial investment costs [3,4].

There are two main techniques to make fish silages, which are the acid silage—made by the addition of a mineral and/or organic acid, or, alternatively, the fermented silage, prepared by anaerobic microbial fermentation [5,6]. Nevertheless, fish silage requires reasonable technological skills and its success depends on the optimization and control of the operating/processing and environmental conditions; namely, fish species as raw-material, type, strength, and amounts of acid(s), microbial strains, and combinations to be used as starter cultures, initial cell density, particle size of raw materials, aerobic to anaerobic cycle, efficiency and maintenance of anaerobic conditions, gas evacuation in the silos, temperature, pH, buffer capacity, and moisture, among others [7]. Fish silage may provide a useful protein source for animal feed, such as swine [8], poultry [9], fish, and ruminants [10,11,12].

In silage processes, the organic acids are formed by microorganisms during fermentation, which lower the pH to values of 3.5–4.5 [13]. Organic acids are generally known as products or metabolites of microbial fermentations. Particularly, under anaerobic conditions the lactic acid bacteria (LAB) may yield (LAB homofermenters) lactic acid as the only metabolite in homofermentative processes or homolactic fermentations, or may yield (facultative or obligate LAB heterofermenters) lactic acid, acetic acid, or ethanol and carbon dioxide in heterofermentative processes or heterolactic fermentations. Lactic and acetic acids, as well as the other organic acids, are known for their antimicrobial and antioxidant features, among others [14]. Besides lactic acid, many strictly and facultative anaerobic microorganisms can yield by fermentation numerous organic acids, such as succinic, acetic, citric, butyric, or propionic acids. For instance, acetic acid bacteria (from *Acetobacter* genus) are important in food fermentations due to the formation of acetic acid from ethanol. High-quality silage is obtained when lactic acid becomes dominant due to its antimicrobial properties and its capacity to drop the pH of the silages very quickly [15].

The production of organic acids in fermented fish silages may represent a biotechnological advantage as it frequently replaces the need of the addition of chemical additives for acidification and, therefore, reduces operating costs [16]. Organic acids are of great importance in food and feed, acting as natural preservatives, and they also may substitute antibiotics. They can be both bacteriostatic and/or bactericidal, and, generally, their effectiveness increases as pH decreases, or in the presence of other antimicrobial factors produced by the microorganisms [17,18]. Lactic and acetic acids produced by LAB play an important role upon growth inhibition of food-borne spoilage, ropiness, and pathogenic microorganisms in fermented food and feed [19,20]. As examples, propionic acid and, mainly, lactic acid at high concentrations exhibited antimicrobial activity against some species of ruminal bacteria [21]. In the presence of organic acids such as acetic, lactic, and succinic acids *Pediococcus acidilactici* K10 demonstrated great in-vitro growth inhibiting ability against the pathogenic strain *Escherichia coli* O157:H7 [22]. Ouattara et al. [23] reported that acetic acid is an organic compound with the greatest growth inhibitory effect against the most common meat spoilage bacteria, followed by propionic, lactic, and citric acids. Knarreborg et al. [24] and Naughton and Jensen [25] found out that the antimicrobial power of several organic acids against coliforms followed the ascending order of propionic < formic < butyric < lactic < fumaric < benzoic acids, while Jensen et al. [26] reported that the growth inhibitory effect of organic acids against *Salmonella typhiumurium*, at pH 4, followed the increasing order of acetic < formic < propionic < lactic < sorbic < benzoic acids. Moreover, from a human nutritional point of view, the organic acids stimulate exocrine pancreatic secretion of enzymes and bicarbonate, which will further aid protein and fat digestion [17,27]. In addition, the terrestrial livestock and shellfish fed with organic acid-supplemented diets disclosed enhanced feed intake, growth-rates, feed utilization effectiveness, and health promoting effects [28,29,30,31].

In the present work, the content of organic acids in acidified and fermented silages undertaken during the three weeks were investigated. The case studies were gibel carp (*Caracius gibelio*) and klunzinger’s ponyfish (*Equulites klunzingeri*) fishes, as well as fish processing by-products. Furthermore, the silages were undertaken with raw-materials under two distinct conditions: in wet and spray-fried forms. The main goals of this research effort were, firstly, to evaluate the potential use of fish-based silages to valorize species of fishes without appreciable or even inexistent commercial value as well as the food wastes resulting from the fish industry activities, and, secondly, to understand the effect of some operating conditions on fish-based silages, particularly the type of silage (acidified and fermented silages), the pre-conditioning treatment of the raw-materials (wet and spray-dried states), and the type of microbial starter cultures in fermented silages (5 LAB strains were assayed).

## 2. Materials and Methods

### 2.1. Feedstocks

Klunzinger’s ponyfish (*Equulites klunzingeri*) and gibel carp (*Carassius gibelio*) fishes were obtained from a local fisherman and sea bass (*Dicentrarchus labrax*) by-products was provided from a Turkish local fish processing industry. The samples were placed in plastic containers (Ferplast, Vicenza, Italy) with ice cuvettes and immediately sent to the laboratory under refrigerated conditions. The samples were further stored at −80 °C before being used for the experimental assays (for 2 weeks).

### 2.2. Microbiological Enumeration, Isolation, and Identification of LAB Strains for the Silage Assays

The LAB strains selected for the fermented silages in this research work were previously isolated and identified. Microbiological procedures were carried out according to previous studies [32] and briefly described here.

#### 2.2.1. Microbiological Enumeration

Aiming at isolating autochthonous LAB strains, 10 g of fish-based samples were suspended, in duplicate, in 90 mL of sterile ringer solution (v/v) (Merck, Darmstadt, Germany) in sterile stomacher bags (LP Italiana SPA, Milano, Italy)), corresponding to the dilution 10^-1^, and aseptically homogenized in a stomacher shaker (IUL, Barcelona, Spain) for 2 min. Then, nine serial decimal dilutions (i.e., 10^−2^ to 10^−10^) were prepared by pipetting under sterile conditions, 1 mL of each previous dilution into 9 mL of sterile ringer solution (Merck, Darmstadt, Germany).

For growth of LAB strains, the culture media was *Lactobacillus* de Man, Rogosa and Sharp agar (MRS), purchased from Merck (Darmstadt, Germany). After rehydration, the pH (WTW 315i, Weilheim, Germany) of the culture media was adjusted to the desired value (at room temperature, ca. 25 °C) with concentrated solutions of NaOH (Merck, Darmstadt, Germany) or HCl (Merck, Darmstadt, Germany) and dissolution was obtained by heating under magnetic agitator (IKA, C MAG HS7, Staufen, Germany). All selective culture media for bacteria were supplemented with 150 mg/mL of cycloheximide (Sigma, Munich, Germany) to prevent growth of yeasts. All culture media were autoclaved (121 °C, 30 min) and after cooling to ca. 45 °C were aseptically poured onto Petri dishes and let to solidify at room temperature.

The cultures were inoculated on MRS in Petri dishes, in triplicate, by the spread plate method with 100 µL of the initial suspension (10^−1^) and the respective decimal dilutions (10^−2^ to 10^−10^) and further incubated. The Petri dishes were divided into 2 quadrants, so as multiple dilutions could be inoculated in a single Petri dish. MRS Petri dishes were further incubated at 30 °C for 3–5 day.

#### 2.2.2. Microbiological Isolation of LAB Strains

After incubation, colonies were randomly isolated according to morphological aspect and frequency of appearance, and purified via adequate sub-culturing—using the quadrant streaking plate method in MRS agar (Merck, Darmstadt, Germany). The purity of the microbial isolates was checked by morphological macroscopic visual appearance of the colonies and microscopic observation of the fresh and Gram-stain cells. Catalase tests were assayed for the isolates. Furthermore, the colonies of non-endospore-forming Gram-positive catalase positive rods and Gram-positive catalase negative or weak rods were selected. Isolates were identified according to the manufacturer’s instructions for the API 50CHL and API 20STREP strip system (BioMereux, Craponne, France). The inoculated strip was incubated for 29 ± 2 °C, for 48 h, and 37 ± 2 °C, for 24 h, respectively. The color reactions were noted as either positive or negative. The result obtained were analyzed using the APILAB PLUS software (BioMereux, Craponne, France).

#### 2.2.3. Microbiological Culture Maintenance

After isolation and purification prior to the microbial identification, the pure strains were kept both on agar slopes (using the same sub-culturing culture media) and cryogenic vials (Nalgen Company, Rochester, NY, USA) with 30% (v/v) sterile aqueous glycerol (Merck, Darmstadt, Germany) and kept at 4 °C. Cultures for the cryogenic vials were obtained by inoculation and incubation in agar slopes.

#### 2.2.4. Molecular Identification of LAB Strains

After the preliminary selection based on the simple phenotype characteristics of microscopic morphology, Gram and catalase and carbohydrate fermentation tests of API, the presumptive LAB strains were subject to the molecular microbial identification to the species level via polymerase chain reaction (PCR) protocols.

For molecular identification of the bacterial strains, 16S rDNA for LAB strains were used. Bacterial stock cultures were reactivated in 5 mL of acetate broth at 37 °C and plasmids isolations were carried out using Gene Jet plasmid extraction kit (Thermo Scientific) according to the manufacturer’s instruction. Isolated plasmids were electrophoresed on agarose gel (1% w/v) and positive bands were visualized on UV transilluminator (Accuris Co., Ltd., USA) and photographed using Olympus C-5060 camera. Isolated plasmid DNA was stored at −20 °C until use. The universal primer set was used for the amplification of approximately 530 bp long fragment [33]. Forward (27 5′-AGAGTTTGATCMTGGCTCCAG-3′) and reverse (519 R 5′ GWA TTA CCG CGG CKG CTG 3′) primers within variable regions in the 16S rRNA encoding genes of isolates were purchased from Inontek (Istanbul, Turkey).

PCR was performed in a DNA thermal cycler (Eppendorf Mastercycler EP Gradient S). PCR was done in 40 μL of reaction mixture containing 1 μL (20 pmol) forward primer, 1μL (20 pmol) reverse primer, 1 μL dNTP (1 mM), 4 μL buffer (NH_4_)_2_SO_4_ (10×), 1.6 μL MgCl_2_, 1 μL DNA polymerase (5 U/μL), 1 μL template DNA (400 ng/mL), and 29.4μL dH_2_O. The products (5 μL plus 1 μL of 6× loading dye) were run on a 1% (w/v) agarose gel. The PCR conditions were conducted as follows: pre-denaturation at 95 °C, for 5 min, followed by 30 cycles of 94 °C, for 1 min, 55 °C, for 50 s, 72 °C, for 1 min, and one cycle of final extension at 72 °C, for 4 min. Following PCR amplification observed fragments (530 bp) were sequenced by Inontek (Istanbul, Turkey). DNA sequences were compared with those in the GenBank database using the BLAST server hosted by the National Center for Biotechnology Information (NCBI, Bethesda, MD, USA).

### 2.3. Preparation of Silages and Sampling

The LAB strains of *Enterococcus gallinarum*, *Lactobacillus brevis*, *Lactobacillus plantarum*, *Pediococcus acidilactici* and *Streptococcus* spp. isolated from fishes and identified in a previous study [32], and briefly described above, were further used for the preparation of fermented silages. Reagents for the silages were of analytical grade. Formic acid (3%, v/v) (Sigma, Munich, Germany) was used to perform acidified silages. Fish-based samples were minced with a refrigerated grinder (3 Φ-mm mesh) (Bursalı Bileyici, Adana, Turkey). Molasses (15%, w_molasses_/w_fish_) as a carbohydrate source and LAB strains at a ratio of 5% (v_LAB_/w_fish_), and giving rise to the initial cell concentration of 10^8^CFU/mL of sterilized distilled water, were independently added to the minced fishes or fish-based by-products. Afterwards, potassium sorbate (2.2 g/kg_sample_) (Sigma, Munich, Germany) and butylated hydroxytoluene (BHT, 250 mg/kg_sample_) (Sigma, Munich, Germany) were added to the previous mixtures as fungicide and antioxidant, respectively. Each fermentation with the distinct LAB starter cultures, as well as with the acidified mixture, were performed in triplicate. In the case of acidified silages, the same procedure was employed but the LAB starter cultures were replaced by the aqueous solution of formic acid 3% (v/w).

The fermented and acidified silages were undertaken at room temperature (*ca*. 27–28 °C) in plastic jars of 3 L. Anaerobic conditions were obtained by the means of jars with tightly fitting lid. The silages were gently stirred by hand for ca. 4–5 min, in a daily basis, until the silage processes were completed, which occurred after three weeks (21 days) of ripening.

After silage and aiming at an effective drying process of the processed fish-based silage samples (here called silage fish-based products), maltodextrin from hydrolyzed corn starch (Alfasol, Turkey) was added to the silages products at a ratio of 1:1 (w/w) before the subsequent drying process. Maltodextrin is a mixture of glycose oligomers added to the silages to ensure the drying process due to the capacity in reducing the stickiness of the silage products. Furthermore, 500 g of silage products from each different silage conditions were subject to spray-drying using a mini spray-dryer (Buchi-290, Flawil, Switzerland). The inlet and outlet temperatures were 160 and 90 °C, respectively, and the aspiration rate and feeding rate were 30 m^3^/h and 20 mL/min, respectively. After spray-drying, the samples were thoroughly mixed and stored at 4 °C after being packed into light-protective plastic bottle, prior to further analysis. The overall experimental design employed is the current research study is summarized in Table 1.

### 2.4. Extraction of Organic Acids

Product samples from silages (Table 1) were subject to the extraction and chromatographic analysis for the separation and quantification of the organic acids. The procedures for the extraction of organic acids were based on the procedures described by Asami et al. [34] with minor modifications. All solvents for extraction of organic acids were purchased from Sigma (Munich, Germany), and were of analytical-reagent grade, HPLC-grade, or pro-analysis-grade.

Briefly, 5 mL of metaphosphoric acid 3% (w/v) was added to 1 g of silage product sample, weighed (Sigma, Munich, Germany) to the milligrams, in 15-mL conic (falcon^®^) tubes, thus giving rise to a solvent to sample ratio in the extraction procedure of 5 mL_solvent_/g_sample_. The sample mixture was homogenized at room temperature, for 1 min, using the Ultra-Turax (T 25 basic IKA-WERKE, Staufen, Germany) at level 2 (9500 1/min), and centrifuged (Hettich 32R, Tuttlingen, Germany) at 5000 rpm for 10 min, at 4 °C. The supernatant (upper liquid layer) was transferred into another 15 mL conic tube and filtered using a Whatman No. 1 filter paper (Maidstone, UK). The filtered extract was transferred to a new round-bottom glass tube, with screw-caps, and stored at 4 °C until further chromatographic analysis of the organic acids were in order.

### 2.5. Separation, Identification, and Quantification of Organic Acids by RP-HPLC-DAD

Solvents for HPLC analysis of organic acids were of HPLC- or superior grade. Standards of organic acids with a purity above 98% (w/w) were purchased from Sigma (Sigma, Munich, Germany). Prior to HPLC analysis, all the samples (organic acid extracts from silage products and standard solutions) were collected into 1 mL-latex-free syringes (Ayset, Turkey) with 0.8 × 38 mm needles (Ayset, Turkey), and further filtered to HPLC vials through 4 mm-disposable PTFE filter media in 0.45 µm-polypropylene housing (Whatman, Kent, UK) adapter.

#### 2.5.1. RP-HPLC-DAD System and Elution Methodology

Lactic, formic, acetic, propionic, and succinic acids were quantified by reversed-phase High-Performance Liquid Chromatography with Diode Array Detector (RP-HPLC-DAD) using the method described by De-Baere et al. [35], with minor modifications. A Shimadzu HPLC system (Kyoto, Japan) equipped with a SPD-M20A diode array detector (DAD), 2 pumps (Shimadzu LC-10AT), auto-sampler (SIL 20AC), column oven (CTO-20AC) and a communication bus module (CBM-20A) was used for the separation and quantification of the organic acids.

A reversed-phase (RP) column Sphereclone^®^ ODS2 C18 (150 mm × 4.6 mm, 5 Φ-mm particle size) was used for the chromatographic separation. The mobile phase for the RP-HPLC-DAD analysis consisted of a binary system of metaphosphoric acid 0.05% (v/v) (Sigma, Munich, Germany) and acetonitrile (Merck, Darmstadt, Germany) according to the elution program previously developed, and optimized [36] as depicted in Table 2. The post-run time of 20 min was employed to re-equilibrate the column and remove any carryover effects. The separation took place at room temperature and the detection was undertaken at wave-lengths (λ) of 210 nm. The extent of separation was calculated via resolution of each two adjacent peaks in the chromatograms.

#### 2.5.2. Preparation of Calibration Curves and Quantification of Organic Acids by RP-HPLC-DAD

Aiming at chromatographic quantification of the organic acids in the fish-based silage products, calibration curves were prepared with the standard of lactic, formic, acetic, propionic, and succinic acids. A standard solution mixture composed by these 5 organic acids with an accurate concentration of 50 mg/mL was prepared in a volumetric flask and dilutions of this stock-standard solution to the final concentrations of 6, 0.6, and 0.06 mg/mL were further accurately prepared in volumetric flasks. The multi-concentration standard mixtures were injected 9 times using the same elution program (Table 3) and RP-HPLC-DAD conditions. The equations of the respective calibration curves, relative standard deviation (RSD), number of standards (*n*) of the organic acids, and reference retention time (RT) are presented in Table 3.

In turn, the extracts of the silage products were injected in triplicate and the organic acids were identified by comparison the retention times of the corresponding standards. Moreover, quantification, using the external standard method, was obtained by integration of the areas of each identified peak, as described above, and the concentration in mg/mL calculated resorting to the corresponding calibration curves (Table 3). The mean values and corresponding standard deviations were calculated from the experimental data obtained from the samples with each distinct silage treatment.

### 2.6. Statistical Analysis

Comparison of different raw-materials (Gibel carp, Klunzinger’s ponyfish and Fish processing by-products) with distinct type of pre-processing conditions (wet and spay-dried) and type of silage (Acidified and fermented with *Enterococcus gallinarum, Lactobacillus brevis, Lactobacillus plantarum, Pediococcus acidilactici* and *Streptococcus* spp.) was independently carried out by applying a one-way ANOVA, using SPSS version 22 (IBM Corporation, New York, NY, USA).

## 3. Results

### 3.1. Organic Acids in Wet Fish-Based Silages

**Figure 1:**
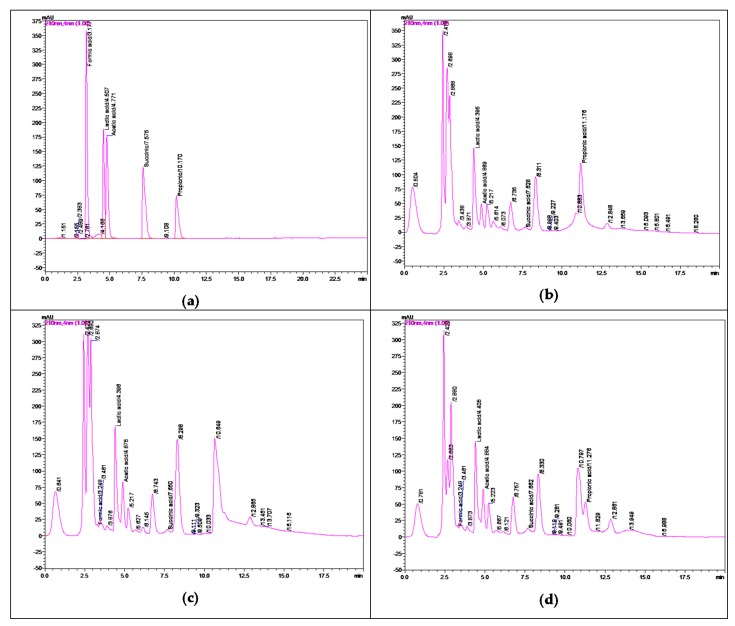
Examples of RP-HPLC-DAD chromatograms of the standards (**a**) of organic acids and organic acids extracted from 3-week silage product samples of (**b**) gibel carp (*Caracius gibelio*), (**c**) klunzinger’s ponyfish (*Equulites klunzingeri*), and (**d**) by-products supplied by a local fish processing company. The chromatographic system and conditions were described above. The elution program for the separation and the calibration curves for quantification of acetic, formic, lactic, propionic and succinic acids were presented in the Table 2; Table 3, respectively.

The Table 4, Table 5 and Table 6 display the organic acid formation (expressed in mg_organic acid_/100 g_sample_) during the 3-week (21-d) wet silages of Klunzinger’s ponyfish (*Equulites klunzingeri*), gibel carp (*Carassius gibelio*), and fish-based processing by-products, respectively. Examples of RP-HPLC-DAD chromatograms obtained during the separation and quantification of organic acids were disclosed in Figure 1.

Formic and propionic acids were not observed in the initial raw fish samples for the wet silages, whereas lactic and acetic were the main organic acids present. Formic, propionic, and lactic acids were the main metabolites produced in acidified wet silages, while lactic and propionic acids were the most abundant organic acids produced throughout the fermented wet silages. Formic acid content in acidified wet silage was in the range from 1051.13 mg/100 g, for klunzinger’s ponyfish, to 1253.89 mg/100 g, for fish processing waste. Formic acid was not produced by any LAB strains in gibel carp wet silage. *P. acidolactici* in klunzinger’s pony fish wet silage and *L. plantarum* in both fish processing waste and klunzinger’s pony fish wet silages did not form formic acid. Apart from these bacteria, formic acid production by other LAB strains were below 4 mg/100 g.

Differences in lactic acid production were statistically significant among fermented wet silage made from gibel carp. Lactic acid formation was the highest with *L. plantarum* (1935. 43 mg/100 g) and *E. gallinarum* (1876.38 mg/100 g) group. Raw fish samples consisted of low succinic acid content (>15 mg/100 g). Among acidified wet silage, the highest succinic acid formation was observed from gibel carp, with value of 152.87 mg/100 g. LAB strains produced statistically similar amount of succinic acid in klunzinger’s pony fish wet silage. In gibel carp and fish processing waste silage, *L. plantarum* generally showed a higher ability to produce succinic acid than other bacteria, although succinic acid formation by *P. acidolactici* and *E. gallinarum* were similar. *Streptococcus* spp. also produced the highest amount of succinic acid in fish processing waste (26.52 mg/100 g).

### 3.2. Organic Acids in Spray-Dried Fish-based Silages

Lactic acid, propionic acid, and acetic acid were the main organic acids found in spray-dried silages (Table 7, Table 8 and Table 9). Formation of formic acid in the acidified spray-dried silages of gibel carp, klunzinger’s pony fish and fish processing waste were 1611.37, 1212.54 and 1005.90 mg/100 g, respectively. LAB strains produced the highest level of formic acid in fish processing waste. Among the fermented spray-dried silages, formic acid production by *L. plantarum* was the lowest (21.98 mg/100 g), although *P. acidolactici* and *E. gallinarum* produced 317.61 and 321.44 mg/100 g of formic acid, respectively. However, in fermented gibel carp spray-dried silage, formic acid formation was only observed in *L. plantarum* group (8.76 mg/100 g).

Lactic acid production in fermented spray-dried silages were above 3200 mg/100 g. Among spray-dried samples, gibel carp silage fermented with *L. plantarum* and *L. brevis* had the highest amount of lactic acid, with respective values of 6003.48 and 5363.48 mg/100 g. Concerning the fermented klunzinger’s pony fish spray-dried silage, no significant differences were observed in lactic acid content among groups apart from *L. brevis*. Gibel carp spray-dried silage fermented with *E. gallinarum* and *Streptococcus* spp. resulted in statistically similar amounts of lactic acid.

Acetic acid production in acidified spray-dried silage varied between 1006.82 and 2030.62 mg/100 g for seafood processing waste. Even if some significant differences (*p* < 0.05) were observed among fermented spray-dried groups in terms of acetic acid production, differences between *P. acidolactici* and *E. gallinarum* groups in acetic acid formation were insignificant. There were also no significant differences (*p* > 0.05) observed between *Streptococcus* spp. and *L. brevis* groups in gibel carp and fermented klunzinger’s pony fish spray-dried silage.

Acidified silages of gibel carp and klunzinger’s pony fish had higher succinic acid contents than that obtained in their fermented spray-dried silages. Klunzinger’s pony fish spray-dried silage and fish processing waste inoculated with *E. gallinarum* showed a significant amount of succinic acid. The highest amount of succinic acid accumulation was attained in gibel carp (134.13 mg/100 g) inoculated with *L. plantarum*, while klunzinger’s pony fish inoculated with *L. brevis* and fish processing waste inoculated with *Streptococcus* spp. unfolded significant amounts of succinic acid (479.69 vs. 465.77 mg/100 g, respectively).

Among spray-dried silages, gibel carp exhibited the highest level of propionic acid. It was also observed that propionic acid accumulated at the highest levels in gibel carp silage (6335.40 mg/100 g) inoculated with *L. plantarum*. Gibel carp spray-dried silage inoculated with *P. acidolactici*, *E. gallinarum* and *Streptococcus* spp. revealed statistically similar amounts of propionic acid. Likewise, fish processing waste silage inoculated with *E. gallinarum* and *Streptococcus* spp. displayed high amount of propionic acid (~5440 mg/100 g). *P. acidolactici* and *L. brevis*. disclosed significantly lower amounts of propionic acid than those achieved by other LAB strains in fish processing waste and klunzinger’s pony fish.

## 4. Discussion

### 4.1. Organic Acids in Wet Fish-Based Silages

The primary technological factor in the production of silages with fish-based raw-materials is the capability of the LAB strains to ferment the raw-materials with the concomitant production of organic acids, chiefly lactic and acetic acids, in order to promote microbial protection of the silages fort further uses, in particular for animal feed [37]. In fermented silages, lactic and propionic acids were primarily formed, while succinic acid was produced in small amounts. Accordingly, Neal-McKinney et al. [38] showed that in a fermented silage, lactic and propionic acids were the main compounds formed, whilst succinic acid was produced the smallest amounts. Moreover, the *Lactobacillus* species formed enough amounts of lactic acid to inhibit the *Camplylobacter jejuniin vitro*. A culture medium containing 100 mM of lactic acid or pH 3.46 (HCl) was able to inhibit completely the development of *C. jejuni* within 1 h [38].

The increase of lactic acid in fermented feed enhances its quality and stability during storage [39], since it prevents the growth of molds and aerobic bacteria [16,40]. In the present study, lactic acid formation was the lowest in raw gibel carp (109.88 mg/100 g) and the highest in raw fish processing waste (138.68 mg/100 g). Lactic acid production was more than 800 mg/100 g in acid silage. In fish processing waste silage, lactic acid production by *L. plantarum*, *P. acidolactici* and *E. gallinarum* were statistically similar, whilst lactic acid production from fermented silage by *Streptococcus* spp. was the highest (1885.46 mg/100 g). Shirai et al. [41] reported that in fermented shrimp waste, containing 5% of glucose and 10% inoculum doses of individual lactic acid bacteria at 30°C for 48 h, lactic acid levels produced by *Lactobacillus* sp. B2, *Lactobacillus casei* A3 and *Lactobacillus pentosus* were 8300, 6300, and 8000 mg/100 g, respectively. In klunzinger’s ponyfish silage, *P. acidolactici* and *E. gallinarum* produced statistically similar and considerable higher lactic acid (1910 mg/100 g) than that obtained in the other groups of microorganisms. Lactic acid production by *L. brevis* in fermented silage was between 1730 and 1885 mg/100 g.

Fermented shrimp waste contained 10% of glucose and 5% of various types of starter cultures had higher than 63 mg lactic acid/g and up to 6 mg acetic acid/g, when samples were allowed to ferment at 30 °C for 48 h [41]. Raw fish processing waste had the lowest amount of acetic acid, although among silage groups, the highest acetic acid formation was observed in the acid silage group of fish processing waste, with a corresponding value of 1022.62 mg/100 g. Wang et al. [42] reported that Italian ryegrass silages inoculated with a commercial *L. plantarum* strain contained a higher ratio of lactic and acetic acids (13.53) than those observed with LAB strains isolated from common vetch, tall fescue, and perennial ryegrass silages undertaken at 25 °C. However, the silages inoculated with *P. acidilactici*, *Lactobacillus paraplantarum,* and *Lactobacillus casei* isolates and developed at 10 °C and 15 °C revealed higher ratios of lactic and acetic acids (8.79, 7.95, and 5.87 for 10 °C; 8.23, 5.87, and 5.52 for 15 °C, respectively) than the silages inoculated with commercial LAB strains [42]. In the current study, the acetic acid in fermented silages was metabolized in the range from 447.15 mg/100 g, in the silage of gibel carp by *Lb. brevis* silage, to 770.64 mg/100 g, in the silage of fish processing waste inoculated by *Streptococcus* spp. Regarding the production of acetic acid, no statistically significant differences (*p* > 0.05) were detected between *P. acidolactici* and *L. brevis*.

From the previous results it was also possible to verify that *Lactobacillus buchneri* (which was used as a starter microbial culture in silage fermentations) anaerobically degraded the lactic acid and did not produce any succinic acid and formic acids [43]. In fermented silages, succinic was the organic acid produced at lowest concentrations after formic acid. Acidified silages revealed the presence of more succinic acid than the fermented silages. Statistically similar amounts of succinic acid were found in klunzinger’s ponyfish silages inoculated with LAB strains. In gibel carp silages, the presence of *L. plantarum* generally resulted in higher amounts of succinic acid than those found with the other LAB. Nevertheless, the formation of succinic acid in the presence of *P. acidolactici* and *E. gallinarum* were similar. The presence of *Streptococcus* spp. also resulted in the highest amounts of succinic acid in fish processing waste (26.52 mg/100 g).

In the present study, raw fish samples did not contain propionic acid, although it was found to be the most highly produced organic acid in fermented and acidified silages, except in the case of klunzinger’s ponyfish silages inoculated with *P. acidolactici* and *E. gallinarum*. *Propionibacterium* are known to produce propionic acid and for their capability to use different carbon sources such as glucose, maltose, sucrose, lactose, lactate, and glycerol. They are also able to utilize complex carbon sources such as hemicelluloses, corncob molasses, and sugarcane molasses, among others [44]. However, and according to Abdul et al. [45], *Propionibacterium freudenreichii* subsp. *Shermanii* produced less propionic acid than the control silage without bacterial inoculation, and statistically similar propionic acid productions were observed by applying *L. plantarum* either alone or in combination with *P. freudenreichii* subsp. *Shermanii* on corn silage. Moreover, propionic acid was produced in all corn silages including in the control silage without bacterial inoculation and was found at the highest concentrations (0.445%, on dry-matter) in the inoculation with *L. plantarum* MA18/5U and *Propionibacterium acidipropionici* MA26/4U (<3 × 10^10^ cfu/g corn) [46]. In the study carried out by Chen et al. [47], it was verified that the silages with no additives (control), with molasses, and with an inoculum (*L. plantarum*) presented propionic acid. In the current study the presence of high levels of propionic acid in acidified and fermented silages are likely to be the result of the presence of other microbiota in the raw-materials and alternative metabolic pathways yielding propionic acid. Moreover, a higher acid content in silages might be attributed to the use of molasses as a carbohydrate source, which apparently stimulated the bacterial fermentation.

Among acidified silage, the highest production of propionic acid was observed in fish processing waste (2149.37 mg/100 g). Amongst fermented fish silage, propionic acid accumulation in gibel carp silage was considerably higher than that obtained with the other fish silage (>2370 mg/100 g). In fermented silages with *L. plantarum* inoculation, the production of propionic acid reached the highest values of 4218.80, 2191.49 and 1372.62 mg/100 g in gibel carp, fish processing waste and klunzinger’s pony fish silages, respectively.

### 4.2. Organic Acids in Spray-Dried Fish-Based Silages

Wagner et al. [48] and Goffin et al. [49] independently found out that the lactic acid firstly formed from glucose was converted to acetic acid after glucose exhaustion in an early stationary growth phase of *Leuconostoc mesenteroides* [48] and *L. plantarum* [49]. In the current study, lactic, propionic and acetic acids were the main organic acids quantified in the spray-dried silages. The strength of lactic acid is relatively high (when compared, for instance, with acetic, propionic and formic acids) and its formation might result in a marked pH decrease, thus preventing synthesis of microbial proteins [21,50,51]. Lactic acid content of spray-dried acid silages were the highest in fish processing waste (4259.58 mg/100 g) and the lowest in klunzinger’s pony fish (1426.59 mg/100 g).

Acetic acid was only produced by *Lactobacillus pentosus* (600 mg/100 g) in a study with fermented shrimp waste [41]. In the current study, all the fermented spray-dried silages contained acetic acid higher than 1200 mg/100 g, whilst the highest value was found in the silage inoculated with *L. plantarum* from gibel carp (1939.35 mg/100 g). Even if some significant changes were observed among fermented spray-dried groups in terms of acetic acid production (*p* < 0.05), the differences between *P. acidolactici* and *E. gallinarum* groups in acetic acid formation were insignificant. There were also no significant differences between *Streptococcus* spp. and *L. brevis* groups (*p* > 0.05) in gibel carp and klunzinger’s pony fish silage.

Succinic acid is known as an intermediate of the tricarboxylic acid cycle and one of the fermentation end-products of anaerobic metabolism. *Anaerobiospirillum succiniciproducens*, *Mannheimia succiniciproducens*, *Actinobacillus succinogenes* and recombinant *Escherichia coli* strains have been reported to be capable of producing a relatively large amount of succinic acid [52,53,54]. Ozcelik et al. [36] also found that LAB strains produced a great amount of succinic acid. In acidified spray-dried silages, the highest amount of succinic acid was formed in klunzinger’s pony fish silage (750.88 mg/100 g), whilst fish processing waste had considerably lower content (37.38 mg/100 g).

Formic and propionic acids are known to be used as preservatives in feeds to prevent microbial degradation [55]. According to Haque et al. [56], propionic acid showed, among all organic acids, the highest growth inhibition efficiency against fungi and yeasts. Propionic acid was one of the main organic acids produced in spray-dried silages. In acidified spray-dried silages, propionic acid concentration ranged from 987.44 mg/100 g for gibel carp to 4928.54 mg/100 g for fish processing waste. Finally, although raw klunzinger’s pony fish and its wet silage inoculated with *P. acidolactici* and *E. gallinarum* did not contain propionic acid, its spray-dried silages was rich in this acid.

## 5. Conclusions

The results of this study successfully showed that fish-based silages in the presence of single starter cultures of lactic acid bacteria (LAB) strains formed noticeable amounts of organic acids including lactic, acetic, and propionic acids during the 3-week (21-d) fermentations. Distinct LAB strains resulted in distinct organic acid profiles in the developed fish-based silages. The experimental data also unfolded the importance of acidification in such processes and the high potential in using LAB starter cultures in fermented silages. With respect to the operational conditions employed in the current experiments, results proved to be adequate for a good development of the 21-day silage processes.

In terms of acid production, the use of wet and spray-dried raw-materials demonstrated distinct performances during fish-based silages, and both cases proved to provide a valuable and safe source for animal and fish feed.

Finally, the results obtained with different fishes (gibel carp and klunzinger’s ponyfish) and fish-based industrial residues (or by-products) also revealed that silage processes are matrix-dependent and evidenced the importance for the optimization and control of the operating/processing conditions on such biotechnological processes. Furthermore, it was also highlighted that the contribution of acidification in the silages by the means of addition of organic acids or through the natural production of organic acids via microbial fermentation (without the need of addition of food preservatives) towards the microbial food and feed safety, quality, and storage preservation of these raw-materials were characterized by an high-nutritional value.

Feed constitutes the operation cost with the greatest impact on livestock and aquaculture production. In addition, non-market fishes and industrial fish-based residues constitute a major environmental problem in a society currently very sensitive to the sustainability of economic activities. Actually, these features represent major technical and socioeconomic bottlenecks that must be overcome through the development of novel and increasingly efficient biotechnological systems. This research study underlined the potential of fish-based (acidified or fermented) silages in favor of the socioeconomic and environmental valorization of such unmarketable fish species and industrial fish-based by-products.

This research represents the first efforts of this multidisciplinary group to explore new insights and develop deeper scientific-technological studies concerning the field of silage, as a promising biotechnology intended for the valorization of products, by-products, residues, and wastes from the fish industry or any other agri-food industrial sector, as well as from agriculture.

## Figures and Tables

**Table 1:** Overall experimental design.

Type of Raw-Material	Type of Preprocessing of Raw-Material	Type of Silage	Organic Acids
- Gibel carp - Klunzinger’s ponyfish - Fish processing by-products	- Wet - Spray-dried	- Acidified fish silage with 3% formic acid - Fermented silage with *Enterococcus gallinarum* - Fermented silage with *Lactobacillus brevis* - Fermented silage with *Lactobacillus plantarum* - Fermented silage with *Pediococcus acidilactici* - Fermented silage with *Streptococcus* spp.	- Acetic acid - Formic acid - Lactic acid - Propionic acid - Succinic acid

**Table 2:** HPLC multistep binary elution gradient used for the separation of organic acids by RP-HPLC-DAD.

Time (min)	%*A*	%*B*	Flow-Rate (mL/min)
0	0	100	0.8
6	0	100	1
9	35	65	0.8
10	2.5	97.5	0.8
15	0	100	0.8
20	0	100	0.8

Eluent ***A***: metaphosphoric acid 0.05% (v/v). Eluent ***B***: Acetonitrile. Stop-time for data collection: 20 min. Post-run time: 20 min.

**Table 3:** Calibration curves of organic acids for the RP-HPLC-DAD analysis.

Organic Acid	Calibration Curve	Linear Correlation Coefficient	*n*	Retention Time (RT) (min)	%RSD
Acetic acid [CH_3_COOH]	*Y* = (2.71176 × 10^−6^) *X*	0.99997	9	4.77	5.414
Formic acid [HCOOH]	*Y* = (2.01452 × 10^−6^) *X*	0.99999	9	3.17	7.206
Lactic acid [CH_3_CH(OH)COOH]	*Y* = (3.57439 × 10^−6^) *X*	0.99995	9	4.50	6.670
Propionic acid [CH_3_CH_2_COOH]	*Y* = (4.33284 × 10^−6^) *X*	0.99999	9	10.17	0.996
Succinic acid [(CH_2_)_2_(COOH)_2_]	*Y* = (2.52321 × 10^−6^) *X*	0.99999	9	7.57	10.169

*n* = Number of standards of the organic acids. *Y* = Concentration of organic acid in mg/mL, *X* = Peak height.

**Table 4:** Yields (mean value ± standard deviation, expressed in mg_organic acid_/100 g_sample_) (*n* = 3) of organic acids in acidified and fermented wet gibel carp (*Caracius gibelio*) silages of 3 weeks. In the fish silages, the acidic conditions were obtained by adding formic acid 3% (v/w), whereas the fermentation were carried out by independent inoculations with pure cultures of distinct lactic acid bacteria (LAB).

Processing Conditions	Acetic Acid	Formic Acid	Lactic Acid	Propionic Acid	Succinic Acid
Raw-material	104.30 ± 9.14	0.00 ± 0.00	109.88 ± 4.14	0.00 ± 0.00	10.09 ± 0.56
Formic acid 3% (v/w)	528.76 ± 26.19	1227.38 ± 17.38 *	1098.22 ± 13.67	1762.34 ± 17.45	152.87 ± 0.21
*Enterococcus gallinarum*	577.73 ± 3.61 ^c^	0.00 ± 0.00	1876.38 ± 13.41 ^d^	2916.82 ± 31.16 ^b^	17.99 ± 0.50 ^c^
*Lactobacillus brevis*	447.15 ± 26.24 ^a^	0.00 ± 0.00	1730.57 ± 19.91 ^c^	2947.25 ± 31.95 ^b^	15.56 ± 0.67 ^b^
*Lactobacillus plantarum*	621.29 ± 2.79 ^d^	0.00 ± 0.00	1935.43 ± 15.98 ^e^	4218.80 ± 5.96 ^d^	19.53 ± 1.02 ^d^
*Pediococcus acidilactici*	471.32 ± 8.75 ^ab^	0.00 ± 0.00	1596.90 ± 6.47 ^b^	3325.93 ± 8.26 ^c^	16.82 ± 0.83 ^bc^
*Streptococcus* spp.	476.75 ± 17.27 ^b^	0.00 ± 0.00	1442.15 ± 8.67 ^a^	2375.95 ± 11.06 ^a^	12.95 ± 1.02 ^a^

The different superscripts (^a^^–e^) in the same column show the statistical differences (*p* < 0.05) observed (*n* = 3) among fermented silage groups. * Formic acid added group.

**Table 5:** Yields (mean value ± standard deviation, expressed in mg_organic acid_/100 g_sample_) (*n* = 3) of organic acids in acidified and fermented wet klunzinger’s ponyfish (*Equulites klunzingeri*) silages of 3 weeks. In the fish silages, the acidic conditions were obtained by adding formic acid 3% (v/w), whereas the fermentation were carried out by independent inoculations with pure cultures of distinct lactic acid bacteria (LAB).

Processing Conditions	Acetic Acid	Formic Acid	Lactic Acid	Propionic Acid	Succinic Acid
Raw-material	173.87 ± 11.65	0.00 ± 0.00	113.41 ± 5.14	0.00 ± 0.00	14.52 ± 0.24
Formic acid 3% (v/w)	584.49 ± 0.88	1051.13 ± 5.16 *	810.23 ± 20.90	1851.06 ± 14.87	43.62 ± 0.65
*Enterococcus gallinarum*	735.88 ± 7.20 ^b^	3.25 ± 0.16 ^b^	1909.39 ± 26.47 ^d^	0.00 ± 0.00	13.29 ± 0.59 ^a^
*Lactobacillus brevis*	697.11 ± 4.23 ^a^	2.76 ± 0.02 ^a^	1848.21 ± 7.05 ^c^	1133.52 ± 19.00 ^a^	12.92 ± 0.66 ^a^
*Lactobacillus plantarum*	674.19 ± 26.09 ^a^	0.00 ± 0.00	1637.14 ± 1.86 ^a^	1372.62 ± 8.24 ^b^	13.78 ± 0.99 ^a^
*Pediococcus acidilactici*	743.23 ± 10.60 ^b^	0.00 ± 0.00	1913.39 ± 30.46 ^d^	0.00 ± 0.00	13.33 ± 0.54 ^a^
*Streptococcus* spp.	752.11 ± 9.33 ^b^	2.90 ± 0.11 ^a^	1751.93 ± 10.97 ^b^	1894.01 ± 17.29 ^c^	12.94 ± 0.74 ^a^

The different superscripts (^a^^–d^) in the same column show the statistical differences (*p* < 0.05) observed (*n* = 3) among fermentation silage groups. * Formic acid added group.

**Table 6:** Yields (mean value ± standard deviation, expressed in mg_organic acid_/100 g_sample_) (*n* = 3) of organic acids in acidified and fermented wet fish processing by-product silages of 3 weeks. In the fish silages, the acidic conditions were obtained by adding formic acid 3% (v/w), whereas the fermentation were carried out by independent inoculations with pure cultures of distinct lactic acid bacteria (LAB).

Processing Conditions	Acetic Acid	Formic Acid	Lactic Acid	Propionic Acid	Succinic Acid
Raw-material	87.23 ± 3.54	0.00 ± 0.00	138.68 ± 2.83	0.00 ± 0.00	11.56 ± 1.03
Formic acid 3% (v/w)	1022.62 ± 55.55	1253.89 ± 22.0 *	2588.74 ± 23.05	2149.37 ± 17.89	35.99 ± 1.66
*Enterococcus gallinarum*	703.10 ± 18.02 ^b^	2.62 ± 0.15 ^a^	1587.69 ± 11.97 ^a^	2080.19 ± 47.39 ^c^	20.79 ± 1.37 ^a^
*Lactobacillus brevis*	770.64 ± 15.38 ^c^	4.03 ± 0.35 ^c^	1885.46 ± 83.41 ^c^	1842.22 ± 25.63 ^b^	26.52 ± 0.87 ^c^
*Lactobacillus plantarum*	674.20 ± 27.19 ^ab^	0.00 ± 0.00	1581.40 ± 21.68 ^a^	2191.49 ± 34.61 ^d^	23.42 ± 1.42 ^b^
*Pediococcus acidilactici*	646.94 ± 17.11 ^a^	3.29 ± 0.19 ^b^	1526.80 ± 38.71 ^a^	960.73 ± 22.48 ^a^	21.22 ± 0.36 ^a^
*Streptococcus* spp.	655.31 ± 3.75 ^a^	2.30 ± 0.16 ^a^	1719.45 ± 9.96 ^b^	2505.18 ± 17.95 ^e^	22.38 ± 0.25 ^ab^

The different superscripts (^a^^–e^) in the same column show the statistical differences (*p* < 0.05) observed (*n* = 3) among fermented silage groups. * Formic acid added group.

**Table 7:** Yields (mean value ± standard deviation, expressed in mg_organic acid_/100 g_sample_) (*n* = 3) of organic acids in acidified and fermented spray-dried gibel carp (*Caracius gibelio*) silages of 3 weeks. In the fish silages, the acidic conditions were obtained by adding formic acid 3% (v/w), whereas the fermentation were carried out by independent inoculations with pure cultures of distinct lactic acid bacteria (LAB).

Processing Conditions	Acetic Acid	Formic Acid	Lactic Acid	Propionic Acid	Succinic Acid
Formic acid 3% (v/w)	1917.20 ± 50.12	1611.37 ± 119.2 *	3958.32 ± 50.35	987.44 ± 84.79	141.74 ± 5.33
*Enterococcus gallinarum*	1361.89 ± 54.27 ^a^	0.00 ± 0.00	4259.89 ± 77.10 ^a^	4311.11 ± 154.52 ^b^	55.53 ± 0.81 ^a^
*Lactobacillus brevis*	1606.32 ± 39.93 ^b^	8.76 ± 0.46	6003.48 ± 131.11 ^d^	3397.95 ± 160.78 ^a^	82.41 ± 3.71 ^b^
*Lactobacillus plantarum*	1939.35 ± 116.34 ^c^	0.00 ± 0.00	5363.48 ± 83.14 ^c^	6335.40 ± 157.98 ^d^	134.13 ± 9.27 ^d^
*Pediococcus acidilactici*	1417.19 ± 65.34 ^a^	0.00 ± 0.00	4705.52 ± 196.59 ^b^	4421.03 ± 116.73 ^bc^	96.95 ± 8.11 ^c^
*Streptococcus* spp.	1580.17 ± 26.41 ^b^	0.00 ± 0.00	4192.19 ± 50.53 ^a^	4569.54 ± 63.80 ^c^	75.12 ± 4.58 ^b^

The different superscripts (^a^^–d^) in the same column show the statistical differences (*p* < 0.05) observed (*n* = 3) among fermented silage groups. * Formic acid added group.

**Table 8:** Yields (mean value ± standard deviation, expressed in mg_organic acid_/100 g_sample_) (*n* = 3) of organic acids in acidified and fermented spray-dried klunzinger’s ponyfish (*Equulites klunzingeri*) silages of 3 weeks. In the fish silages, the acidic conditions were obtained by adding formic acid 3% (v/w), whereas the fermentation were carried out by independent inoculations with pure cultures of distinct lactic acid bacteria (LAB).

Processing Conditions	Acetic Acid	Formic Acid	Lactic Acid	Propionic Acid	Succinic Acid
Formic acid 3% (v/w)	2030.62 ± 31.62	1212.54 ± 10.8 *	1426.59 ± 13.99	1595.92 ± 30.25	750.88 ± 46.49
*Enterococcus gallinarum*	1359.12 ± 36.85 ^a^	253.99 ± 13.91 ^b^	3647.40 ± 79.28 ^b^	2605.53 ± 138.81 ^e^	454.29 ± 16.11 ^d^
*Lactobacillus brevis*	1564.00 ± 24.81 ^b^	18.59 ± 0.81 ^a^	3431.80 ± 99.60 ^a^	411.78 ± 0.55 ^a^	479.69 ± 21.48 ^e^
*Lactobacillus plantarum*	1602.10 ± 19.60 ^b^	301.32 ± 2.57 ^c^	3975.00 ± 64.29 ^c^	1247.41 ± 27.50 ^b^	66.85 ± 3.16 ^b^
*Pediococcus acidilactici*	1408.05 ± 42.66 ^a^	26.51 ± 1.98 ^a^	3755.22 ± 143.65 ^b^	2063.86 ± 89.28 ^d^	346.46 ± 10.48 ^c^
*Streptococcus* spp.	1579.27 ± 114.73 ^b^	27.71 ± 0.70 ^a^	3805.97 ± 71.29 ^bc^	1530.73 ± 27.41 ^c^	38.80 ± 2.11 ^a^

The different superscripts (^a^^–e^) in the same column show the statistical differences (*p* < 0.05) observed (*n* = 3) among fermented silage groups. * Formic acid added group.

**Table 9:** Yield (mean values ± standard deviation, expressed in mg_organic acid_/100 g_sample_) (*n* = 3) of organic acids in acidified and fermented spray-dried fish processing by-product silages of 3 weeks. In the fish silages, the acidic conditions were obtained by adding formic acid 3% (v/w), whereas the fermentation were carried out by independent inoculations with pure cultures of distinct lactic acid bacteria (LAB).

Processing Conditions	Lactic Acid	Formic Acid	Acetic Acid	Propionic Acid	Succinic Acid
Formic acid 3% (v/w)	4259.58 ± 61.93	1005.90x ± 22.75^y^ *	1006.82 ± 20.38	4928.54 ± 88.20	37.38 ± 1.61
*Enterococcus gallinarum*	3987.31 ± 77.38 ^c^	321.44 ± 10.49 ^d^	1574.57 ± 14.14 ^c^	5464.99 ± 96.47 ^d^	308.51 ± 3.93 ^c^
*Lactobacillus brevis*	4036.68 ± 99.09 ^c^	290.46 ± 7.26 ^c^	1833.95 ± 20.18 ^d^	3779.11 ± 41.57 ^b^	25.93 ± 0.83 ^b^
*Lactobacillus plantarum*	4218.92 ± 24.03 ^d^	21.98 ± 0.87 ^a^	1370.63 ± 35.40 ^b^	4292.87 ± 89.12 ^c^	18.15 ± 0.94 ^ab^
*Pediococcus acidilactici*	3780.18 ± 16.50 ^b^	317.61 ± 14.80 ^d^	1587.63 ± 5.09 ^c^	474.66 ± 24.33 ^a^	12.80 ± 1.41 ^a^
*Streptococcus* spp.	3259.16 ± 45.33 ^a^	217.81 ± 4.02 ^b^	1289.77 ± 17.11 ^a^	5421.72 ± 19.83 ^d^	465.77 ± 12.97 ^d^

The different superscripts (^a^^–e^) in the same column show the statistical differences (*p* < 0.05) observed (*n* = 3) among silage groups. * Formic acid added group.
